# Supplementary material for: MetaRibo-Seq measures translation in microbiomes
Source: Nat Commun. 2020 Jun 29;11:3268. doi: 10.1038/s41467-020-17081-z (PMC7324362; doi:10.1038/s41467-020-17081-z)
Supplement: Supplementary file 10 — Supplementary Data 7 [file 41467_2020_17081_MOESM10_ESM.zip › File2/Confidence_High_Taxonomy/319564_out.krona.html]

Javascript must be enabled to view this page.

members
magnitude
magnitudeUnassigned
count
unassigned
taxon
rank

319564\_out

244

superkingdom
244
1
2

SRS1055034\_contig\_number\_contig-100\_34832.34833

1
phylum
74201

1
203494
class

order
48461
1

1647988
family
1

1
239934
genus


SRS075773\_contig\_number\_26002
1
239935
species

1239

SRS021219\_contig\_number\_contig-100\_23379.23379SRS023346\_contig\_number\_contig-100\_3266.50711SRS024388\_contig\_number\_contig-100\_15402.50068SRS097958\_contig\_number\_contig-100\_2128.147324SRS1041141\_contig\_number\_6846SRS104912\_contig\_number\_7970SRS140513\_contig\_number\_contig-100\_23939.62504SRS893285\_contig\_number\_contig-100\_8276.8277
242
phylum
8

species
2292898
1

SRS048164\_contig\_number\_32232

2
91061
class

186826
order
2

81852
family
1

1
1350
genus

1
species
1352

SRS016840\_contig\_number\_contig-100\_19707.19708

1
33958
family

1
1578
genus


SRS014573\_contig\_number\_6276
1
species
47770


SRS016095\_contig\_number\_24941SRS893300\_contig\_number\_12395SRS893358\_contig\_number\_5031
1263012
species
3

227
186801
class

186802

SRS017821\_contig\_number\_contig-100\_1726.196626SRS018351\_contig\_number\_contig-100\_13527.48422SRS024492\_contig\_number\_contig-100\_30441.72440SRS063040\_contig\_number\_43550SRS142781\_contig\_number\_contig-100\_2320.92893SRS143895\_contig\_number\_contig-100\_2227.265634SRS148424\_contig\_number\_contig-100\_395.186017
order
227
7

2
family
150

SRS018984\_contig\_number\_contig-80\_925.238793SRS140492\_contig\_number\_12412
186803

2
189330
genus


SRS143991\_contig\_number\_contig-100\_754.165649
1
1896999
species

1
88431
species

SRS014979\_contig\_number\_contig-100\_969.190632


SRS045004\_contig\_number\_28259
1952109
species
1


SRS058070\_contig\_number\_30841SRS097920\_contig\_number\_contig-100\_596.193376SRS144183\_contig\_number\_37299
3
742723
species

841

SRS011302\_contig\_number\_23863SRS144135\_contig\_number\_1645SRS148970\_contig\_number\_1884
genus
23
3

4
2049040
species

SRS017103\_contig\_number\_1808SRS019161\_contig\_number\_3818SRS1055049\_contig\_number\_5947SRS1055076\_contig\_number\_5873

166486
species
8

SRS014923\_contig\_number\_24584SRS015264\_contig\_number\_19816SRS045739\_contig\_number\_2141SRS056519\_contig\_number\_18494SRS098073\_contig\_number\_6822SRS1041037\_contig\_number\_contig-100\_1139.83388SRS893342\_contig\_number\_3032SRS893378\_contig\_number\_23445


SRS063518\_contig\_number\_736SRS146764\_contig\_number\_6075SRS148253\_contig\_number\_34SRS893259\_contig\_number\_6018SRS893288\_contig\_number\_1137
species
360807
5

species
2293138
1

SRS015065\_contig\_number\_34178

1
1262941
species

SRS149879\_contig\_number\_12659


SRS053335\_contig\_number\_contig-100\_248.98977
1
species
301301

4
genus
572511

1
species
40520

SRS015431\_contig\_number\_39663


SRS042628\_contig\_number\_38853
species
765821
1

species
33039
1

SRS148784\_contig\_number\_contig-100\_718.168806

1
species
33038

SRS018656\_contig\_number\_7012

species
1898203
3

SRS056259\_contig\_number\_13521SRS076976\_contig\_number\_20619SRS077024\_contig\_number\_14078

genus
33042
6

4
species
1078091

SRS019496\_contig\_number\_contig-100\_146.113036SRS062701\_contig\_number\_5907SRS065504\_contig\_number\_36019SRS077454\_contig\_number\_contig-100\_39.119812

1
410072
species

SRS077730\_contig\_number\_contig-100\_601.121581


SRS020233\_contig\_number\_57695
species
116085
1

207244
genus
1

1
649756
species

SRS013965\_contig\_number\_34880

1769710
genus
1

species
1653434
1

SRS051882\_contig\_number\_contig-100\_380.136916

1
2109691
species

SRS053398\_contig\_number\_133

103
39491
species

SRS011239\_contig\_number\_contig-100\_1230.167243SRS012969\_contig\_number\_39117SRS013940\_contig\_number\_2331SRS014313\_contig\_number\_5713SRS014683\_contig\_number\_contig-100\_833.120461SRS014948\_contig\_number\_contig-100\_1108.127444SRS015486\_contig\_number\_297SRS015694\_contig\_number\_12036SRS015794\_contig\_number\_contig-100\_931.90765SRS015854\_contig\_number\_23252SRS015960\_contig\_number\_contig-100\_2971.101385SRS016267\_contig\_number\_contig-100\_1437.78896SRS016495\_contig\_number\_contig-100\_1208.119317SRS016989\_contig\_number\_10944SRS017433\_contig\_number\_contig-100\_1669.102390SRS017701\_contig\_number\_21084SRS018313\_contig\_number\_contig-100\_675.86829SRS018623\_contig\_number\_15252SRS019267\_contig\_number\_contig-100\_479.63702SRS019445\_contig\_number\_18596SRS019685\_contig\_number\_contig-100\_694.231578SRS020869\_contig\_number\_contig-100\_612.229189SRS021484\_contig\_number\_15702SRS022071\_contig\_number\_36503SRS022137\_contig\_number\_20865SRS022524\_contig\_number\_16303SRS023526\_contig\_number\_contig-100\_227.235522SRS024132\_contig\_number\_contig-100\_560.253500SRS043768\_contig\_number\_contig-100\_1046.186362SRS044535\_contig\_number\_contig-100\_183.147114SRS045195\_contig\_number\_contig-100\_9512.51182SRS045826\_contig\_number\_contig-100\_1353.130718SRS046068\_contig\_number\_640SRS046717\_contig\_number\_4378SRS047044\_contig\_number\_11444SRS047433\_contig\_number\_21766SRS048060\_contig\_number\_13575SRS048870\_contig\_number\_contig-100\_1327.129204SRS049959\_contig\_number\_contig-100\_826.279823SRS050422\_contig\_number\_37057SRS050925\_contig\_number\_contig-100\_513.193314SRS050998\_contig\_number\_contig-100\_902.102591SRS052697\_contig\_number\_44597SRS053214\_contig\_number\_contig-100\_821.131576SRS054956\_contig\_number\_contig-100\_1145.97799SRS055982\_contig\_number\_6921SRS057478\_contig\_number\_4540SRS058723\_contig\_number\_contig-100\_842.51661SRS063190\_contig\_number\_11881SRS063370\_contig\_number\_11777SRS065397\_contig\_number\_12767SRS075021\_contig\_number\_contig-100\_428.61025SRS075984\_contig\_number\_contig-100\_1464.150761SRS076804\_contig\_number\_82SRS077194\_contig\_number\_32945SRS077294\_contig\_number\_contig-100\_1095.124980SRS077335\_contig\_number\_contig-100\_736.56907SRS077641\_contig\_number\_contig-100\_1402.117057SRS078176\_contig\_number\_21805SRS097889\_contig\_number\_contig-100\_781.199719SRS098061\_contig\_number\_contig-100\_221.80183SRS098644\_contig\_number\_43071SRS098717\_contig\_number\_20859SRS104311\_contig\_number\_44823SRS104327\_contig\_number\_3671SRS104400\_contig\_number\_60656SRS104975\_contig\_number\_4226SRS105153\_contig\_number\_8542SRS1054716\_contig\_number\_10636SRS1055022\_contig\_number\_24227SRS1055056\_contig\_number\_13144SRS142599\_contig\_number\_32100SRS142890\_contig\_number\_44190SRS142980\_contig\_number\_contig-100\_24924.24925SRS143070\_contig\_number\_16203SRS143342\_contig\_number\_contig-100\_736.138948SRS143466\_contig\_number\_26436SRS143523\_contig\_number\_contig-100\_860.153116SRS143598\_contig\_number\_16890SRS143780\_contig\_number\_contig-100\_1171.177122SRS144362\_contig\_number\_43150SRS144506\_contig\_number\_contig-100\_589.313311SRS144537\_contig\_number\_44699SRS144603\_contig\_number\_10749SRS144714\_contig\_number\_contig-100\_943.173325SRS145497\_contig\_number\_contig-100\_354.126327SRS146813\_contig\_number\_957SRS147271\_contig\_number\_contig-100\_2356.224277SRS147425\_contig\_number\_9896SRS147919\_contig\_number\_contig-100\_1462.118329SRS148091\_contig\_number\_2851SRS148721\_contig\_number\_55249SRS148874\_contig\_number\_contig-100\_1939.89722SRS149075\_contig\_number\_19551SRS149181\_contig\_number\_31282SRS149244\_contig\_number\_contig-100\_2085.74594SRS149784\_contig\_number\_contig-100\_609.143770SRS893170\_contig\_number\_9177SRS893231\_contig\_number\_7383SRS893292\_contig\_number\_2210SRS893295\_contig\_number\_contig-100\_786.68945SRS893341\_contig\_number\_contig-100\_893.89446SRS971275\_contig\_number\_26840

541000
family
3

3
1263
genus

165186
species
2

SRS050026\_contig\_number\_contig-100\_2326.74983SRS147346\_contig\_number\_12011


SRS019968\_contig\_number\_9472
species
40518
1

31979
family
10


SRS053649\_contig\_number\_13400
1485
1
10
genus


SRS051031\_contig\_number\_35363
1262785
species
1


SRS023715\_contig\_number\_11424
1
species
2293054


SRS062654\_contig\_number\_contig-100\_926.155886SRS076929\_contig\_number\_contig-100\_725.152728SRS1054691\_contig\_number\_20486SRS147022\_contig\_number\_13300SRS147139\_contig\_number\_35775SRS147766\_contig\_number\_9210
6
59620
species

1
2292311
species

SRS143417\_contig\_number\_32874

family
186806
57

genus
1730
57


SRS011084\_contig\_number\_contig-100\_653.214393SRS011405\_contig\_number\_14479SRS013158\_contig\_number\_4750SRS013476\_contig\_number\_contig-100\_1604.212520SRS013951\_contig\_number\_contig-100\_197.239378SRS014235\_contig\_number\_contig-100\_1034.267459SRS014287\_contig\_number\_20013SRS014459\_contig\_number\_contig-100\_1750.199834SRS014613\_contig\_number\_7066SRS014855\_contig\_number\_14260SRS015190\_contig\_number\_contig-100\_699.101632SRS015578\_contig\_number\_5651SRS015579\_contig\_number\_contig-100\_1037.151206SRS015890\_contig\_number\_contig-100\_456.146849SRS016018\_contig\_number\_contig-100\_1280.92274SRS016203\_contig\_number\_6346SRS016335\_contig\_number\_49710SRS016954\_contig\_number\_contig-100\_1397.144034SRS017191\_contig\_number\_18560SRS017521\_contig\_number\_contig-100\_158.214668SRS018817\_contig\_number\_contig-100\_1234.124976SRS019030\_contig\_number\_contig-100\_884.119898SRS019068\_contig\_number\_55786SRS019178\_contig\_number\_4863SRS019381\_contig\_number\_8997SRS019397\_contig\_number\_contig-100\_868.87233SRS019601\_contig\_number\_28888SRS022713\_contig\_number\_contig-100\_2044.23679SRS024331\_contig\_number\_contig-100\_782.180974SRS042284\_contig\_number\_7203SRS043701\_contig\_number\_contig-100\_1417.101618SRS045645\_contig\_number\_31689SRS049712\_contig\_number\_contig-100\_964.140284SRS050752\_contig\_number\_contig-100\_893.174271SRS054905\_contig\_number\_contig-100\_571.154309SRS063985\_contig\_number\_contig-100\_348.174952SRS064276\_contig\_number\_contig-100\_819.225541SRS064276\_contig\_number\_contig-100\_819.225541SRS075821\_contig\_number\_contig-100\_1410.121320SRS077231\_contig\_number\_6403SRS077502\_contig\_number\_contig-100\_1048.108644SRS077552\_contig\_number\_contig-100\_380.175782SRS098571\_contig\_number\_contig-100\_2245.336436SRS098881\_contig\_number\_contig-100\_2041.158680SRS104485\_contig\_number\_5141SRS142503\_contig\_number\_52363SRS142542\_contig\_number\_contig-100\_721.166363SRS143876\_contig\_number\_contig-100\_405.236788SRS146812\_contig\_number\_contig-100\_60.238027SRS147377\_contig\_number\_contig-100\_1549.73306SRS147977\_contig\_number\_contig-100\_1419.73149SRS148159\_contig\_number\_55293SRS148196\_contig\_number\_43927SRS148319\_contig\_number\_contig-100\_2404.81373SRS149325\_contig\_number\_contig-100\_1024.100474SRS893252\_contig\_number\_20018SRS893270\_contig\_number\_contig-100\_836.135269
species
142586
57


SRS015663\_contig\_number\_contig-100\_826.174723
2292885
species
1
